# Coronary Plaque Characteristics Assessed by 256-Slice Coronary CT Angiography and Association with High-Sensitivity C-Reactive Protein in Symptomatic Patients with Type 2 Diabetes

**DOI:** 10.1155/2016/4365156

**Published:** 2016-08-08

**Authors:** Jinling Zhang, Zhehao Lv, Deli Zhao, Lili Liu, Yong Wan, Tingting Fan, Huimin Li, Ying Guan, Bailu Liu, Qi Yang

**Affiliations:** ^1^Department of CT, The Second Affiliated Hospital of Harbin Medical University, Harbin 150086, China; ^2^Department of Radiology, Xuanwu Hospital, Beijing 100053, China; ^3^Biomedical Imaging Research Institute, Cedars Sinai Medical Center, Los Angeles, CA 90048, USA

## Abstract

Little is known regarding plaque distribution, composition, and the association with inflammation in type 2 diabetes mellitus (DM2). This study aimed to assess the relationship between coronary plaque subtypes and high-sensitivity C-reactive protein levels. Coronary CTA were performed in 98 symptomatic DM2 patients and 107 non-DM2 patients using a 256-slice CT. The extent and types of plaque as well as luminal narrowing were evaluated. Patients with DM2 were more likely to have significant stenosis (>50%) with calcified plaques in at least one coronary segment (*p* < 0.01); the prevalence rates of diffuse calcified plaques in the DM2 and non-DM2 groups were 31.6% and 4.7%, respectively (*p* < 0.01). Plasma hs-CRP levels in DM2 with calcified plaques were higher compared with values obtained for the non-DM2 group (*p* < 0.01). In conclusion, combination of coronary CTA and hs-CRP might improve risk stratification in symptomatic DM2 patients.

## 1. Introduction

Diabetes mellitus is the most important risk factor for coronary artery disease (CAD). Type 2 diabetes mellitus (DM2) has reached epidemic proportions globally, affecting populations of both developed and developing countries [1]. CAD is often asymptomatic in DM2 patients until the onset of myocardial infarction or sudden cardiac death [2]. It has been widely demonstrated that acute cardiac events are related to rupture and acute thrombosis caused by a mildly stenotic plaque, namely, the vulnerable plaque [3]. Major advances in CAD prevention require early detection of the vulnerable plaques.

Conventional X-ray coronary angiography is the current gold standard for invasive evaluation of CAD. However, it only shows the lumen of the vessel, greatly underestimating the atherosclerosis burden. A noninvasive assay to directly detect coronary atherosclerosis would therefore be beneficial. Coronary CTA provides comprehensive information noninvasively regarding the location, severity, and characteristics of coronary atherosclerotic plaques: noncalcified, calcified, and mixed plaques can be identified. A previous study showed that vulnerable plaques in diabetic patients tend to occur at multiple sites, with high atherosclerotic burden [4].

Inflammation is a fundamental component of atherosclerosis [5, 6]. The most widely tested inflammatory biomarker is high-sensitivity C-reactive protein (hs-CRP), which predicts the risk of a first MI in healthy individuals and future coronary events in patients with stable CAD [7]. It is well known that both elevated CRP and specific plaque subtypes are associated with poor disease outcome [8, 9]. Understanding how CRP and vulnerable plaques are related and using imaging techniques to assess this relationship may enable the early identification of vulnerable patients. Recently, in a cohort of asymptomatic subjects, increased CRP levels were found to be associated with the prevalence of any plaque and mixed calcified plaques, as well as significant coronary stenosis [10]. To the best of our knowledge, no study has been reported regarding symptomatic diabetic patients. Therefore, we aimed to assess the relationship between hs-CRP levels and plaque subtypes in symptomatic patients with DM2 using 256-slice CT.

## 2. Methods

### 2.1. Study Population and Design

From December 2013 to December 2014, 205 patients underwent 256-slice CT coronary angiography, including 98 DM2 cases and 107 individuals without DM2. They were 108 men and 97 women, 48 ± 15 years old, with BMI of 22.15 ± 2.36 kg/m^2^. No patient had a known previous CAD. Serum hs-CRP levels were measured before coronary CTA. The diagnostic criteria for DM2 were based on WHO guidelines.

Exclusion criteria were heart rate ≥ 90 bpm, atrial fibrillation, severe renal insufficiency (serum creatinine > 120 *μ*mol/L), severe respiratory insufficiency, hyperthyroidism, and allergy to iodine-based contrast. The study was approved by the Institutional Review Board of the hospital, and informed consent was obtained from each patient.

### 2.2. Instruments, Equipment, and Scanning Method

CTA examinations were performed on a 256-slice CT (Brilliance iCT; Philips, Amsterdam, Netherlands) and a power injector (SCT-211; Medrad Inc., Indianola, PA, USA). The scan protocols were as follows: detector width, 80 mm; detector collimation, 128 × 0.625 mm; slice acquisition, 128 × 0.625 mm using a *z*-flying focal spot; gantry rotation time, 0.27 s. Tube voltage and current were set at 300–500 mAs per rotation and 120–140 kVp, respectively. The contrast material (Ultravist Solution 370 mg I/mL; Bayer Healthcare, Berlin, Germany) was intravenously injected through an antecubital vein using a 20-gauge needle connected to a power injector (SCT-211; Medrad Inc., Indianola, PA, USA). Contrast material injection timing was controlled by the bolus-tracking technique in the ascending aorta (signal attenuation threshold 100–120 Hounsfield units [HU]). Data acquisition was initiated with a mean delay of 6 s after reaching the threshold in the ascending aorta. A total amount of 60–80 mL of contrast material was injected at 5 mL/s followed by 30 mL of saline chaser. The mean effective radiation dose of the coronary CTA was 1.58 ± 0.36 mSv.

### 2.3. Coronary CTA Images Analysis

All scans were analyzed independently by two experienced radiologists blinded to the clinical information, on a Brilliance workstation (Philips Healthcare, Amsterdam, Netherlands). Each lesion was identified using the multiplanar reconstruction technique and free mode maximum intensity projection.

**Figure 1:**
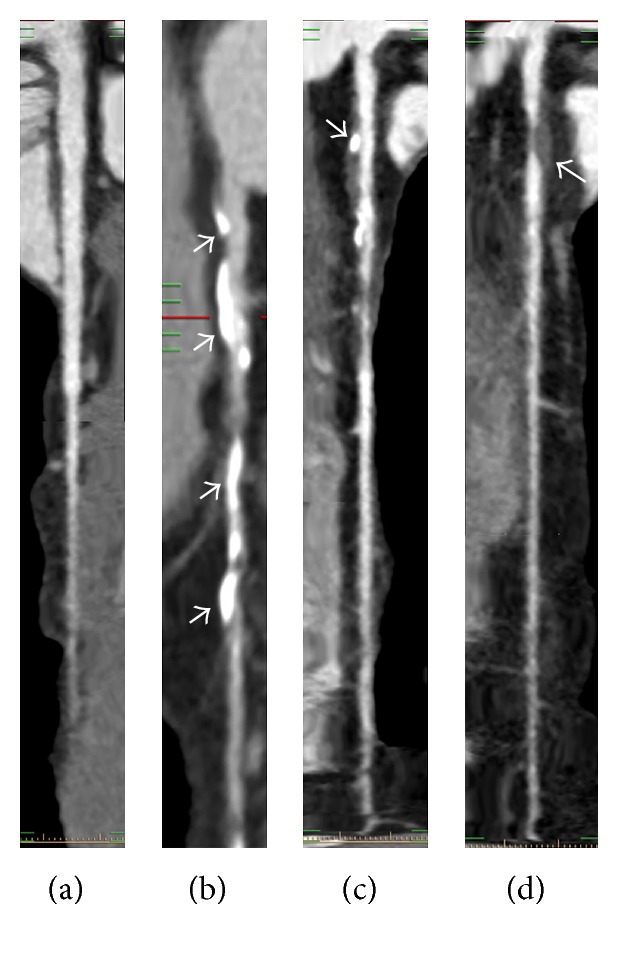
Representative CCTA images showing the assessed plaque subtypes. (a) Normal; (b) calcified arterial plaques; (c) mixed calcified arterial plaque; and (d) noncalcified arterial plaques.

The 15 coronary segments were defined according to American Heart Association (AHA) standards. Lesions were classified by the maximal luminal stenosis observed on any plane; grading of stenosis was further classified as normal appearing (<25%) and mild (25%–49%) and moderate (50%–74%) and severe (≥75%) narrowing. Plaques occupied by calcified tissue >50% of the plaque area (density > 130 Hounsfield units in native scans) were classified as calcified arterial plaques (CAP); those with <50% calcium were considered mixed calcified arterial plaques (MCAP); and plaques without calcium were classified as noncalcified arterial plaques (NCAP) as previously described (Figure 1).

### 2.4. Statistical Analysis

Continuous variables are mean ± standard deviation (SD) and categorical variables as number and percentage. The extent and types of plaque as well as luminal narrowing were evaluated and compared between diabetic and nondiabetic patients. Groups were compared using Student's *t*-test and Chi-square test. The patients were further divided into 3 groups according to median hs-CRP levels: low/normal group (hs-CRP ≤ 1 mg/L), intermediate group (1 mg/L < hs-CRP ≤ 2 mg/L), and high group (>2 mg/L). Multivariate logistical regression analysis was performed to explore the relationship between diabetes, CRP, and type and extent of plaque. Statistical analyses were performed with the SPSS 18.0 software (SPSS, Chicago, IL, USA). *p* < 0.05 was considered statistically significant.

## 3. Results

### 3.1. Baseline Characteristics

The patients were 48 ± 15 years old and included 52.7% men; body mass index values were 22.15 ± 2.36 kg/m^2^. The average DM2 duration was 8.5 ± 7.8 years. Compared with subjects without DM2, no significant differences in the levels of total cholesterol, triglyceride, high-density lipoprotein cholesterol, and low-density lipoprotein cholesterol were obtained.  Table 1 summarizes the general characteristics of the study population.

### 3.2. Coronary Artery Plaque Subtypes and Distribution

A total of 432 coronary vessels (4.3 ± 0.18 per patient) and 820 segments (8.2 ± 0.06 per patient) had plaques. In total, 1194 plaques (5.97 ± 0.22 per patient) were detected. Figure 1 depicts the three coronary plaque subtypes found in this study. Calcified plaques (54.7%) were more frequently detected than mixed or noncalcified ones (*p* < 0.001). The most commonly affected coronary vessel was the LAD artery in both groups: 40.4% LAD, 27.1% RCA, 18.3% LCX, and 14.2% LM were diseased in diabetics, with 39.0% LAD, 32.1% RCA, 18.2% LCX, and 10.7% LM in nondiabetic patients (all *p* < 0.001).

### 3.3. DM2 Patients versus Non-DM2 Patients

262 diseased coronary vessels with 453 affected segments were found in diabetic patients, whereas 170 diseased coronary vessels and 367 affected segments were observed in nondiabetic individuals. Diffuse vessel disease was more commonly detected in DM2 patients than nondiabetic subjects (31.6% versus 4.7%; *p* < 0.01). In addition, more diseased segments were found in patients with diabetes (8.23 ± 4.48) compared with nondiabetic subjects (3.67 ± 2.42, *p* < 0.05). Furthermore, CAD tended to be more severe in DM2 patients as both left main/LAD coronary artery and multivessel diseases were more frequently diagnosed, although the difference was not statistically significant. More calcified plaques and less noncalcified plaques were detected in diabetics compared with nondiabetic patients, respectively (72.9% versus 48.1%, *p* < 0.001; 27.1% versus 51.9%, *p* < 0.001). As DM2 disease time increased from 5 to 10 and 10 to 15 years, the proportion of calcified plaques increased, with that of noncalcified plaques decreasing significantly (63.1% versus 24.0%, *p* < 0.001). Obstructive plaques were more abundant in diabetic patients compared with nondiabetics (28.3% versus 7.6%, *p* < 0.05). The plaque burden and stenosis data are shown in Table 2.

### 3.4. Prevalence of Coronary Plaques Based on CRP

**Figure 2:**
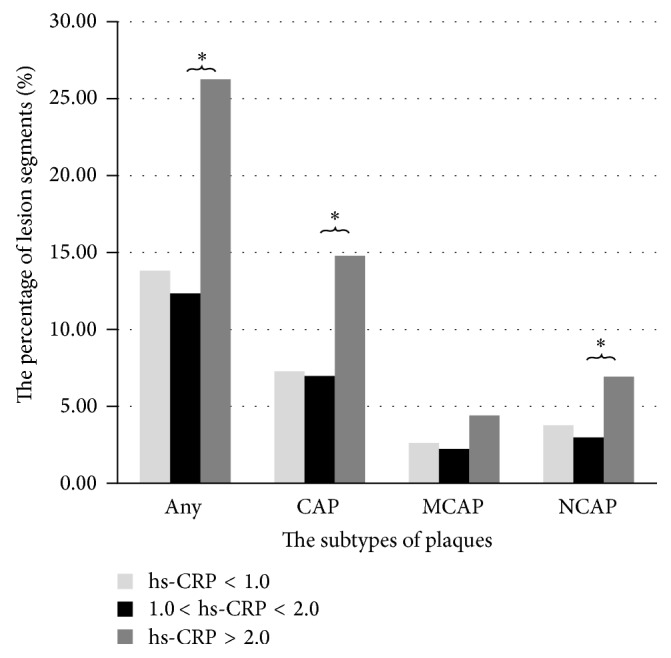
Percentage of patients with various plaque subtypes according to CRP category. Subjects with high hs-CRP levels were more likely to have any plaque, CAP, or NCAP compared with the second and third tertiles. *∗* refers to a statistically significant association between hs-CRP and the subtype of plaques.

**Figure 3:**
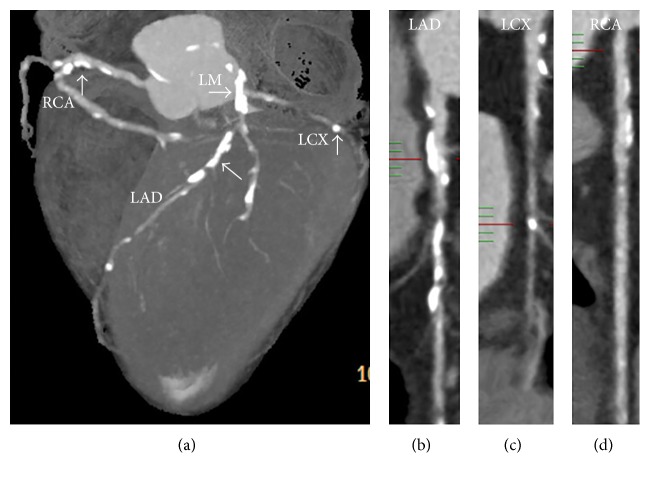
A 62-year-old man with 17 years' history of diabetes with multivessel disease. (a) Volume rendering image depicts unsmooth edges for left and right coronary vessels. (b), (c), and (d) show diffuse calcified plaques and multiple stenosis distributed in the whole course of the LAD, LCX, and RCA arteries (arrows).

Figure 2 depicts the prevalence of any coronary plaque subtype according to the various CRP cutoffs. Subjects with high CRP levels were more likely to have any plaque, CAP, or NCAP compared with individuals showing normal CRP levels (*p* < 0.01). In contrast, no significant difference was obtained for MCAP. Plasma CRP levels were significantly higher in individuals with DM2 compared with controls (3.232 ± 0.327 mg/L versus 1.937 ± 0.198 mg/L, *p* < 0.01).  Figure 3 shows a symptomatic DM2 patient with increased CRP, in whom coronary CTA revealed triple vessel disease with diffuse calcified plaques.

### 3.5. Relationship between Diabetes, CRP, and Type and Extent of Plaque

Both the unadjusted and the multivariable logistic regression analyses for the presence of any coronary plaque and plaque subtype are listed in Table 3. Subjects were divided into groups according to CRP levels, and all analyses were performed using the low-normal CRP group as the reference category. Subjects with high CRP were observed to be at increased risk for the presence of CAP in the unadjusted values (odds ratio [OR], 2.761; 95% confidence interval [CI], 2.673 to 5.893) and the adjusted model 1 (odds ratio [OR], 3.056; 95% confidence interval [CI], 1.494 to 6.228). No difference was observed in the risk for NCAP or MCAP for the unadjusted values. When examining the presence of a specific plaque subtype, subjects with CRP had no increased risk for the presence of any type of plaque for the adjusted values.

## 4. Discussion

In the present study, differences in coronary plaque characteristics and plasma hs-CRP levels between symptomatic DM2 patients and nondiabetics were observed. We also demonstrated that elevated hs-CRP levels were associated with increased risk for plaque formation, including CAP and NCAP.

Significantly more diffuse CAP were present in symptomatic DM2 patients compared with nondiabetics, indicating that the presence of diffused calcium is associated with greater atheroma burden, in agreement with previous reports [4, 11]. Traditionally, calcified plaque is considered an established, stable, and quiescent atheroma. However, in several cross-sectional studies in patients with acute coronary syndrome, spotty calcification is associated with culprit lesions [12–14]. Multiple studies also demonstrated that the presence of coronary artery calcification in asymptomatic individuals is a predictor of future cardiac events [15–17]. Clearly, plaque calcification represents a dynamic process related to oxidized lipids and inflammatory activity. The natural history of atherosclerosis is considered a dynamic process varying from early lesion development to more advanced plaques. In our study, we demonstrate that elevated levels of CRP are associated with increased risk for the presence of any plaque and CAP, but not NCAP or MCAP; patients with symptomatic DM2 were more likely to have significant stenosis with CAP in at least one coronary segment. Our findings may indicate a more rapid atherosclerosis development in diabetics, with a faster progression from noncalcified to completely calcified lesions.

Coronary CTA provides data regarding the coronary tree and atherosclerotic plaques beyond simple luminal narrowing and plaque type defined by calcium content [18]. Compared with calcium score analysis, coronary CTA data sets provide submillimeter isotropic spatial resolution, and the possibility of CT attenuation based tissue characterization enables the quantification of total coronary plaque burden and individual plaque components. If the high-risk plaques reported in this study are indeed important for prognosis, coronary CTA would be a potential candidate screening tool, since it might identify silent vulnerable plaques not otherwise detected by functional imaging.

Chronic inflammation plays a major role in all phases of atherosclerosis [9], and DM2 presence and development are associated with subclinical systemic inflammation [19]. In this study, we found that severe stenosis rates and hs-CRP levels were significantly higher in symptomatic DM2 patients. Also, nearly half of the plaques caused obstructive stenosis in symptomatic DM2 patients. It has been clearly demonstrated that both elevated hs-CRP levels and specific plaque subtypes are associated with poor cardiovascular outcomes [10]. Therefore, people at risk should pay more attention to their blood CRP levels. Our findings may help understand how hs-CRP and vulnerable plaques are related, also showing that coronary CTA enables the assessment of such relationship.

Our study has several limitations. First, it remains unclear whether coronary calcification predicts plaque instability or is merely a marker of plaque burden. More animal studies are warranted for better understanding of calcification mechanisms. Second, because we categorized coronary plaque subtypes into NCAP, MCAP, and CAP as opposed to quantifying in a continuous manner the burden of calcified and noncalcified plaque components, there is a chance of misclassification. Finally, although our analysis reflects the relationship between diffused calcification and elevated levels of hs-CRP in patients with diabetes, the resultant impact on clinical outcome remains to be determined. Future studies are required to determine the prognostic role of this methodology in patients with DM2.

In conclusion, combination of coronary CTA and hs-CRP might incrementally improve risk stratification in DM2 patients. Future prospective studies are needed to establish the association between the presence of CAP and acute coronary events.

## Figures and Tables

**Table 1:** Basic characteristics.

Characteristic	All patients	DM2 patients	Non-DM patients
Number (*n*)	205	98	107
Age (years)	48 ± 15	46 ± 14	44 ± 13
Male (%)	108 (52.7)	55 (56.1)	48 (44.9)
BMI (kg/m^2^)	22.15 ± 2.36	23.22 ± 1.37	22.43 ± 2.14
Mean heart rate	65 ± 13	64 ± 11	63 ± 12
Hypertension (%)	131 (63.9)	78 (79.6)	53 (49.5)
Current smoking (%)	82 (40.0)	40 (40.8)	42 (39.3)
Family history of coronary disease (%)	116 (56.6)	55 (56.1)	61 (57.0)
TC (mmol/L)	4.82 ± 1.12	4.85 ± 1.09	4.86 ± 1.14
TG (mmol/L)	2.18 ± 0.81	2.09 ± 1.02	1.91 ± 0.78
HDL-C (mmol/L)	1.24 ± 0.28	1.19 ± 0.23	1.34 ± 0.26
LDL-C (mmol/L)	2.96 ± 0.45	2.92 ± 0.63	2.94 ± 0.46

Data are mean ± SD or *n* (%). BMI: body mass index; TC: total cholesterol, TG: triglyceride, HDL-C: high-density lipoprotein cholesterol, and LDL-C: low-density lipoprotein cholesterol.

**Table 2:** Comparison of plaque burden and grading of stenosis between diabetic and nondiabetic patients.

	All patients	DM2	Non-DM	*p* value
Plaque burden	1194	826	368	
Calcified plaque^*∗∗*^	653 (54.7%)	476 (57.6%)	177 (48.1%)	<0.01
Noncalcified plaque	330 (27.6%)	207 (25.1%)	123 (33.4%)	0.021
Mixed calcified plaque	211 (17.7%)	143 (17.3%)	68 (18.5%)	0.046
Grading of stenosis				
Normal appearing (<25%)	2180 (72.8%)	1047 (69.8%)	1133 (75.5%)	<0.01
Mild narrowing (25%–49%)	563 (18.7%)	287 (19.1%)	276 (18.4%)	0.042
Moderate narrowing (50%–74%)	181 (6.0%)	106 (7.1%)	75 (5.0%)	0.039
Severe narrowing^*∗∗*^ (≥75%)	76 (2.5%)	60 (4.0%)	16 (1.1%)	<0.01
Nonobstructive plaques	765 (64.1%)	485 (40.6%)	281 (23.5%)	0.028
Obstructive plaques^*∗*^	429 (35.9%)	338 (28.3%)	91 (7.6%)	<0.05

^*∗*^ *p* < 0.05; ^*∗∗*^ *p* < 0.01.

**Table 3:** Multivariate logistical regression analysis of coronary plaque subtype and CRP level.

Presence of coronary plaque	Unadjusted	Model 1	Model 2
	OR (95% CI)	OR (95% CI)	OR (95% CI)
CAP			
Low/normal CRP	1	1	1
Intermediate CRP	1.564 (1.454, 3.284)	2.067 (1.239, 3.961)	1.365 (0.633, 3.925)
High CRP	2.761 (2.673, 5.893)	3.056 (1.494, 6.228)	2.964 (0.129, 4.852)
MCAP			
Low/normal CRP	1	1	1
Intermediate CRP	2.182 (0.715, 6.661)	2.304 (0.739, 7.186)	3.094 (0.678, 14.124)
High CRP	2.727 (0.436, 17.046)	2.266 (0.340, 15.105)	9.043 (0.898, 98.318)
NCAP			
Low/normal CRP	1	1	1
Intermediate CRP	0.397 (0.126, 1.254)	0.422 (0.131, 1.363)	0.358 (0.089, 1.435)
High CRP	0.436 (0.707, 2.727)	0.300 (0.042, 2.143)	2.068 (0.032, 2.255)

Model 1: adjusted for age and sex.

Model 2: adjusted for age, sex, smoking, low-density lipoprotein cholesterol, high-density lipoprotein cholesterol, total cholesterol, triglycerides, body mass index, and DM history.
